# Editorial: The importance of the central hemodynamic in the cardiovascular diseases development

**DOI:** 10.3389/fcvm.2022.1036440

**Published:** 2022-10-10

**Authors:** Jose Fernando Vilela-Martin, Pedro Jose Forcada

**Affiliations:** ^1^Internal Medicine Department, State Medical School in São José Do Rio Preto (FAMERP), São Paulo, Brazil; ^2^University of Buenos Aires, Buenos Aires, Argentina; ^3^Non-Invasive Vascular Laboratory of CardioArenales, Universidad Austral, Buenos Aires, Argentina

**Keywords:** central hemodynamic parameters, arterial stiffness, hypertension, pulse wave velocity (PWV), target organ damage

Before Christ, Egyptian and Chinese scholars described the importance of arterial pulse wave (1). The evolution of knowledge about the arterial system during the past centuries led to the development of the peripheral blood pressure (BP) measurement in 1896 (2). This assessment generated all the knowledge that guides the hypertension diagnosis and treatment until the current days. However, data on BP continues to be centered on peripheral measures.

In the last 20 years, countless pathophysiological, epidemiological, and pharmacodynamic studies have shown the participation of the central hemodynamic parameters and, more specifically of the arterial stiffness, in the development of cardiovascular disease (3). Arterial stiffness constitutes an important independent factor that can predict future risk of cardiovascular events, it is an important indicator of vascular changes, and it is associated with cardiovascular mortality in various groups of patients with cardiometabolic diseases as well as health population (4–8). Therefore, understanding the etiologic role of the arterial damage in the development of cardiovascular disease and its participation as risk marker for evolution of the preclinical atherosclerotic process is very important for research of the new diagnostic ways and management of vascular damages. Thus, this Research Topic “*The Importance of the Central Hemodynamic in Cardiovascular Diseases Development*” sought to attract relevant research on the subject. A brief internet search on it found 6,311 articles published in the PUBMED database with the terms “arterial stiffness and hypertension” and more than 11,000 papers with the terms “central hemodynamic and hypertension”. Thus far, four articles have been published on this important topic about the relationship between central hemodynamic and cardiovascular diseases (9).

Firstly, Zhang et al. evaluated non-invasive parameters of central hemodynamics and vascular function. Initially, the systemic hemodynamic index (SHI), measured by impedance cardiography, evaluated hemodynamic data (stroke volume, heart rate, cardiac output, cardiac index, etc.). In the sequence, the vascular function was evaluated using the flow-mediated dilation and brachial-ankle pulse wave velocity (cfPWV), which represented the vascular damage index (VDI). They found that the SHI, considered as a surrogate marker, presented a negative correlation with VDI and suggested that SHI/VDI score may work as practical tool for vascular risk stratification.

The second paper, Plunde et al. studied predictive factors of the arterial stiffness in individuals submitted to aortic valve surgery. They demonstrated that cardio ankle vascular index (CAVI) is less dependent of the peripheral blood pressure, and it presents lower interobserver variation in the cases of aortic valve disease, a fact that may be explained because CAVI measures the arterial stiffness from a greater proportion of the arterial network (which includes peripheral segments) compared to cfPWV measure. In addition, they demonstrated that arterial stiffness may be underestimated in cases of aortic valve disease, and the post-operative stiffness is a better indicator of the real vascular status compared to pre-surgery situation.

The next two publications approach the relationship between central hemodynamic parameters and target organ damage (TOD) (Hu et al.; Chao et al.). Hu et al. studied the association of TOD with central and peripheral BP using the non-invasive 24-h ambulatory BP monitoring (ABPM) in hypertensive subjects with cardiovascular risk factors. They evaluated the carotid intima-media thickness (IMT) and/or carotid plaque, left ventricular hypertrophy (LVH), and kidney alterations [urine albumin/creatinine and/or estimated glomerular filtration rate (eGFR)] as TOD. The prevalence of TOD was 47.3% for elevated IMT, 25.6% for LVH, and 20.5% for microalbuminuria, these values are closed to those found by our group in hypertensive patients followed up in a specialized outpatient clinic (10). They also demonstrated that peripheral BP obtained by 24 h monitoring exhibited better correlation with TOD than office BP and 24 h central BP, this data was also found in a Spanish study (11). Nevertheless, the prognostic value of 24-h central BP for estimating 10-year cardiovascular risk was higher than 24-h peripheral BP (Hu et al.).

Therefore, although the measurement of office BP is recommended by the guidelines, we need to consider that it does not provide complete information regarding the involvement of target organs, since the BP obtained by ABPM presents a better correlation with TOD than the office BP in hypertensive patients. Moreover, measurements performed outside the office, whether by ABPM or residential BP monitoring, provide us with greater substrates for therapeutic decisions which focused on cardiovascular protection and prognosis (12, 13).

Lastly, Chao et al. assessed the risk of TOD (LVH, IMT, eGFR, and urine albumin/creatinine ratio) in individuals with different phenotypes of peripheral BP and cfPWV. Similarly, the prevalence rates of TOD were close to other studies (10, 14, 15). They found that the increase in cfPWV or elevation in peripheral systolic BP alone had different effects on TOD. For example, higher peripheral BP presented increased risk of microalbuminuria, while elevated cfPWV led to greater risk of LVH. Somehow, this data shows that TOD may be influenced by many factors, and not just BP or cfPWV. In a recent study carried out in a Brazilian hypertensive population, this association with other factors became evident (10). It demonstrated that age, microalbuminuria, triglycerides, high density lipoprotein cholesterol, and diabetes and the presence of carotid disease were related to TOD. Moreover, only nocturnal BP (by ABPM) was correlated with LVH (10). As previously described, the correlation between central BP and TOD was not better than peripheral BP (Hu et al.). However, this should not preclude the use of central hemodynamics for a better evaluation of the hypertensive patient. In our view, the use of central hemodynamic parameters is intended to promote subject management in an individualized way, in such a way that the diagnosis and its treatment are more accurate and more adequate.

Therefore, as observed, central hemodynamic parameters provide an assessment of cardiovascular risk and are associated with the presence of TOD and can be used as biomarkers (16). Additionally, the evaluation of central hemodynamic can be particularly useful in other situations, such as:

1. To differentiate spurious isolated systolic hypertension (false hypertension) from true hypertension in young hypertensive individuals.
2. To detect early vascular aging, allowing early intervention and CV risk reduction in middle-aged adults and, finally,
3. The cfPWV may explain the higher arterial pulsatility and rigidity and be a prognostic marker and a therapeutic purpose in the elderly.

## Author contributions

All authors listed have made a substantial, direct, and intellectual contribution to the work and approved it for publication.

## Funding

JV-M is the recipient of a fellowship from Brazilian National Council for Scientific Technological Development (CNPq, BPQ) (grant number #313715/2021-1) and he thanks FAPESP for the grant support (2016/08203-6).

## Conflict of interest

The authors declare that the research was conducted in the absence of any commercial or financial relationships that could be construed as a potential conflict of interest.

## Publisher's note

All claims expressed in this article are solely those of the authors and do not necessarily represent those of their affiliated organizations, or those of the publisher, the editors and the reviewers. Any product that may be evaluated in this article, or claim that may be made by its manufacturer, is not guaranteed or endorsed by the publisher.
